# Optoelectronic Properties of Monolayer Hexagonal Boron Nitride on Different Substrates Measured by Terahertz Time-Domain Spectroscopy

**DOI:** 10.3390/nano10040762

**Published:** 2020-04-16

**Authors:** Muhammad Bilal, Wen Xu, Chao Wang, Hua Wen, Xinnian Zhao, Dan Song, Lan Ding

**Affiliations:** 1 Key Laboratory of Materials Physics, Institute of Solid State Physics, Chinese Academy of Sciences, Hefei 230031, China; mbilal@mail.ustc.edu.cn (M.B.); cwang@theory.issp.ac.cn (C.W.); hwen@theory.issp.ac.cn (H.W.); xnzhao@theory.issp.ac.cn (X.Z.); dsong@theory.issp.ac.cn (D.S.); 2 Key Laboratory of Materials Physics, Institute of Solid State Physics, University of Science and Technology of China, Hefei 230026, China; 3 School of Physics and Astronomy and Yunnan Key Laboratory for Quantum Information, Yunnan University, Kunming 650091, China; dinglan@ynu.edu.cn

**Keywords:** monolayer, hexagonal boron nitride, chemical vapor deposition, terahertz, time domain spectroscopy

## Abstract

Monolayer (ML) hexagonal boron nitride (hBN) is an important material in making, e.g., deep ultraviolet optoelectronic and power devices and van der Waals heterojunctions in combination with other two-dimensional (2D) electronic systems such as graphene and ML MoS${}_{2}$. In this work, we present a comparative study of the basic optoelectronic properties of low resistance ML hBN placed on different substrates such as SiO${}_{2}$/Si, quartz, PET, and sapphire. The measurement is carried out by using terahertz (THz) time-domain spectroscopy (TDS) in a temperature regime from 80 to 280 K. We find that the real and imaginary parts of the optical conductivity obtained experimentally for low resistance ML hBN on different substrates can fit well to the Drude–Smith formula. Thus, we are able to determine optically the key sample and material parameters (e.g., the electronic relaxation time or mobility, the carrier density, the electronic localization factor, etc.) of ML hBN. The effect of temperature on these parameters is also examined and analyzed. The results obtained from this study enable us to suggest the appropriate substrate for ML hBN based electronic and optoelectronic devices. This work is relevant to the application to a newly developed 2D electronic system as advanced electronic and optoelectronic materials.

## 1. Introduction

Hexagonal boron nitride (hBN) has emerged as a realm of great interest for advanced electronic and optoelectronic devices, because of its distinguishable characteristics such as high chemical and thermal stability, mechanical strength, low dielectric constant, and near-zero polarization [1]. hBN has a very wide band gap, which makes it important for ultraviolet (UV) and neutron detectors, transparent membranes, UV LEDs, dielectric layers, etc. [2]. More interestingly, monolayer (ML) hBN has been realized recently [3]. The most important feature of ML hBN is that the electronic band gap is found to be about 6.07 eV [4], which is the widest value found among two-dimensional (2D) electronic materials discovered and developed so far. Such an extraordinary feature, combined with ultra-high chemical stability and excellent thermal conduction, makes ML hBN the material for emerging applications such as deep UV optoelectronic devices [5] and power devices [6]. Moreover, the proton transport has been investigated where the significant increase in proton conductivity can be observed when hBN is reduced to a monolayer [7]. The value of proton conductivity through ML hBN (about 100 mS cm^−2^) was found to be much higher compared to ML MoS${}_{2}$ or graphene (about 5 mS cm^−2^). The electric transport through mono- and multi-layer hBN has also been examined [8]. It was found that the electron transport through the graphite/hBN/graphite heterostructure was also increased significantly when hBN was reduced to a monolayer. Hence, ML hBN has high conducting values compared to bulk or multi-layer hBN. More importantly, in recent years, ML hBN has been applied for the realization of van der Waals heterojunctions in combination with other 2D electronic systems such as graphene [9] and transition metal dichalcogenide (TMD) based 2D electronic systems [10]. ML hBN has been used as a dielectric substrate for graphene based electronic devices, owing to its unique characteristics like thermal stability and as an insulator [11]. Encapsulation of ML MoS${}_{2}$ by hBN has drastically altered the electronic and optical responses of ML MoS${}_{2}$ because of the hybridization of the electronic states between MoS${}_{2}$ and hBN [12]. For example, through the measurements of temperature dependent and time resolved photoluminescence (PL), it has been found that the momentum forbidden dark excitons can be observed with energy lower than 83 meV in an ML MoS${}_{2}$/hBN heterojunction. In recent years, the investigation of ML hBN based van der Waals heterojunctions has become a hot and fast-growing field of research in electronics and optoelectronics [13].

It is known that the high quality free-standing ML hBN is normally weakly conducting [14]. Similar to conventional semiconductor based devices, ML hBN based electronic and optoelectronic devices are often placed on the substrate. Generally, the substrate can affect the performance of the device through proximity effects. The presence of the substrate can introduce the long- and short-range charged disorders caused by chemical bonding or interface roughness and introduce the extra phonon scattering from the substrate, which can influence the carrier density, mobility, and dielectric screening of the conducting carriers in ML hBN [15]. More specifically, ML hBN is an in-plane hexagonal crystal with uniaxial symmetry. In such a crystal system, Rashba spin-orbit coupling (SOC) often exists [16,17]. When ML hBN film is placed on a substrate, the presence of the heterostructure can lead to an inversion symmetry-breaking field along the direction normal to the 2D plane of ML hBN [18,19]. As a result, the Rashba SOC in ML hBN can be further enhanced by the presence of the substrate [20]. Furthermore, it is found that the presence of the dielectric substrate can induce the van der Waals force and the exchange interaction in the heterostructure [21]. Therefore, it is of great importance and significance to examine the effect of the substrate on the key sample and material parameters of ML hBN, and this becomes the prime motivation of the present study.

For the investigation of the electronic and optoelectronic properties of an electronic material, an optical experiment is one of the most popularly employed techniques because the measurement normally does not require the fabrication of the contacting electrodes on the sample. In particular, terahertz (THz) time-domain spectroscopy (TDS) has been a powerful optical technique in characterizing and studying the optoelectronic properties of electronic materials and devices [22]. The major advantage of the THz TDS measurement is that the real and imaginary parts of the optical conductivity can be measured directly without involving the Kramers–Kronig (K-K) transformation, and the electron density can be determined in the absence of a magnetic field. Very recently, we measured the key sample and material parameters such as electronic relaxation time, carrier density and electronic localization factor in ML MoS${}_{2}$ placed on different commonly used substrates [23] and in La${}_{0.33}$Pr${}_{0.34}$Ca${}_{0.33}$MnO${}_{3}$ thin films [24] by using THz TDS measurements. Using this technique, we have also examined the Faraday rotation effect and obtained the effective electron masses in bi- and tri-layer graphene in the presence of a magnetic field [25]. In this study, we generalize the THz TDS technique we used previously to the investigation of the optoelectronic properties of ML hBN.

## 2. Samples and Experimental Measurements

### 2.1. Sample Growth

In this study, ML hBN samples were prepared by using the standard techniques for film growth and transfer. ML hBN was grown on a high quality copper foil by chemical vapor deposition (CVD) at a growth temperature of 1000 °C [15,26], where the borane ammonia complex (BH${}_{6}$N) was taken as the precursor material to grow ML hBN. Ammonia borane has a melting temperature of about 106 °C, and it decomposes into hydrogen, ammino borane (BH${}_{2}$NH${}_{2}$), and borazine(HBNH${}_{3}$). Aminoborane is a white crystalline solid and stable at room temperature, while borazine is in gas form. Borazine is the main building block of hBN. During growth, borazine was subjected to a copper substrate whose temperature was already maintained at 1000 °C inside the furnace. Borazine oxidized to hexagonal boron nitride on the copper foil at 1000 °C. We employed the standard seal transfer method [27,28] to place the ML hBN film onto the target substrates such as SiO${}_{2}$/Si, quartz, PET, and sapphire. After the synthesis of ML hBN on the copper foil, ML hBN was coated with polymethyl methacrylate (PMMA) and the copper foil etched away. Acetone was applied to clean the residues from the ML hBN induced during the etching process. Then, the ML hBN film was transferred onto different substrates.

The monolayer of the hBN film was identified and confirmed by the color and contrast of the film via optical microscopy images [15]. We found that the hBN layers on different substrates were ML continuous films and had high crystal quality. It should be noted that during the CVD growth of the ML hBN films in this study, some nitrogen vacancies could be present in hBN films, similar to the case pointed out and measured for hBN epilayers grown by MOCVD [29]. These nitrogen vacancies can play the role of deep-level (about 2.4 eV) impurities and significantly affect the optical and excitonic properties of hBN epilayers [29]. Due to the presence of the N-vacancies, we found that the ML hBN films on the above-mentioned substrates were p-type, similar to ML hBN samples grown through the CVD technique by other groups [5,29] and that the ML hBN films on these substrates had relatively low electric resistance. The areal size of the sample was about 1 cm × 1 cm. The thicknesses of the sapphire, quartz, and SiO${}_{2}$/Si (with 300 nm silicon oxide on silicon) substrates were 0.35 mm, 1 mm, and 0.55 mm, respectively. The sample surface was very clean, and the surface color was very uniform.

### 2.2. THz TDS Measurement System

In this study, THz TDS technique [23] was applied to measure the transmission of the THz light beam through ML hBN on different substrates, in studying the corresponding optoelectronic properties. Transmission signals through each sample (hBN/substrate) and through the corresponding bare substrate were measured separately. The measurements were carried out in the temperature regime from 80 to 280 K. The details of the measurement and the data treatment were similar to our very recent work on ML MoS${}_{2}$ [23]. The incident THz light beam was transmitted vertically through the sample surface and the strength of the transmitted THz field measured. In the THz TDS system, the femtosecond (fs) fiber laser (ROI optoelectronics) generated 1550 nm wavelength laser pulses with an 80 fs pulse duration at a 100 MHz repetition rate. The fs laser beam was divided into two beams. The higher intensity beam was the pump (generating) light, which was irradiated on an InGaAs photoconductance antenna (PCA. Melno, Germany) to generate THz pulses. The generated THz beam was a horizontally polarized field, which was directed towards the sample. The sample was fixed on a sample holder in the cryostat (ST-500, Jains) with a quartz window, and the sample chamber was in a vacuum. Since the cryostat had a quartz window and quartz can only transmit efficiently up to 1.2 THz [30], in the measurement, we used the THz frequency ranging from 0.2 THz to 1.2 THz. The THz light focused on the sample was linearly polarized along the 2D-plane of ML hBN. The lower intensity fs laser beam was the probe (detecting) beam with a time delay introduced by a displacement platform, which was radiated on another InGaAs PCA. This beam was used to detect the transmitted THz field through the sample by photo-electric sampling. In the present study, the variation of the temperature was down to liquid nitrogen temperature at about 80 K. Thus, we were able to measure the strength of the THz electric field transmitted through a sample as a function of the delay time in a temperature range from 80 K to 280 K. Moreover, the experimental system was slowly flushed with dry nitrogen gas to prevent the absorption of the THz waves by moisture in free-space.

## 3. Results and Discussion

**Figure 1:**
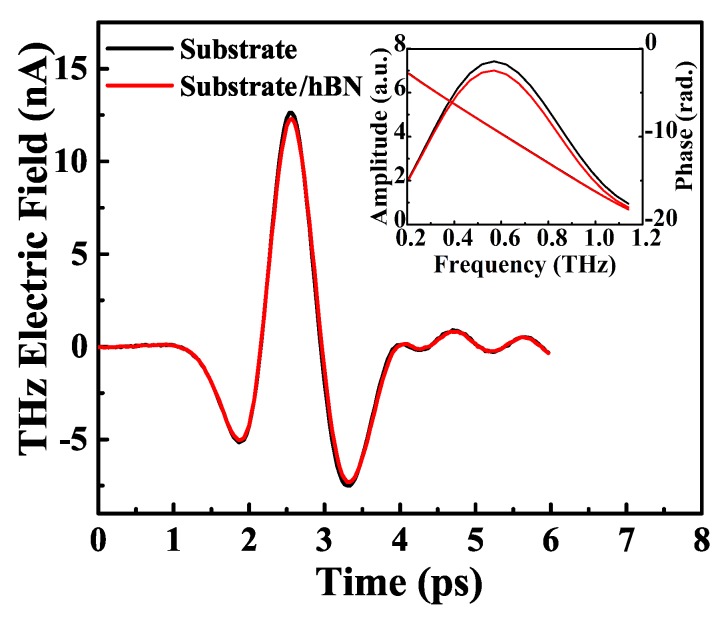
The THz electric field strength transmitted through ML hBN on the quartz substrate (red curve) and through the bare quartz substrate (black curve), respectively, as a function of delay time at T = 280 K. The inset shows the corresponding amplitudes and phase angles of the THz electric field strengths transmitted through the ML hBN sample and the substrate in the frequency domain. The results for the phase angles coincide roughly.

**Figure 2:**
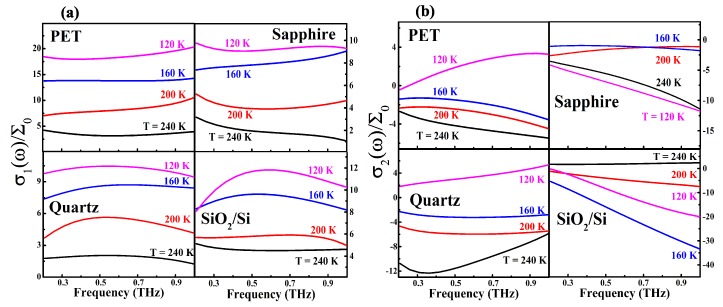
(**a**) Real $\sigma_{1}\left(\omega \right)$ and (**b**) imaginary $\sigma_{2}\left(\omega \right)$ parts of the optical conductivity as a function of radiation frequency f=ω/2π at different temperatures for ML hBN on different substrates as indicated. Here, $\Sigma_{0}=e^{2}/4\hbar =6.07\times 10^{-5}$ S.

**Figure 3:**
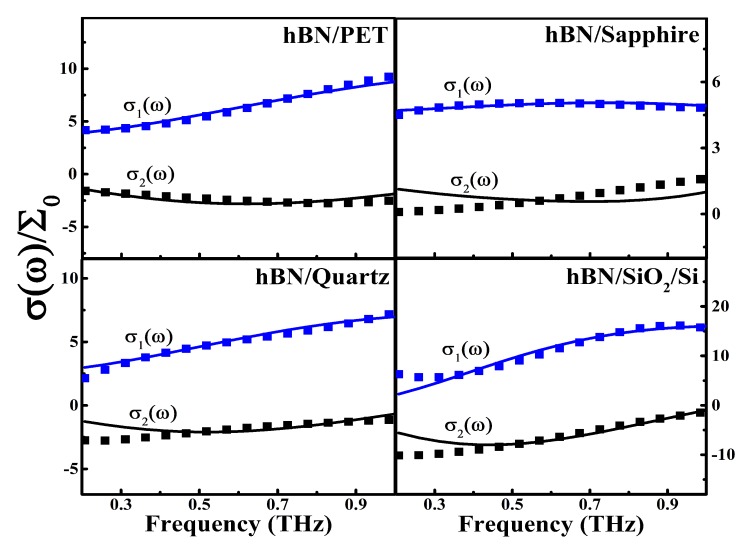
The experimental and fitted (through Drude–Smith formula) real $\sigma_{1}\left(\omega \right)$ and imaginary $\sigma_{2}\left(\omega \right)$ parts of optical conductivity as a function of radiation frequency f=ω/2π for ML hBN on PET, sapphire, quartz, and SiO${}_{2}$/Si substrates at 280 K, respectively. Here, the solid curves are obtained from the Drude–Smith formula, and the dots are experimental data. $\Sigma_{0}=e^{2}/4\hbar$.

By using THz TDS, we could measure the electric field strength of the THz light beam transmitted through a sample (hBN/substrate), $E_{sample+substrate}\left(t\right)$, and through a substrate, $E_{substrate}\left(t\right)$, in the time-domain (see Figure 1). After Fourier transforming of these measured data, we could obtain the corresponding electric field strengths for a sample (hBN/substrate), $E_{sample+substrate}\left(\omega \right)$, and a substrate, $E_{substrate}\left(\omega \right)$, in the frequency-domain. The amplitudes and the phase angles of $E_{sample+substrate}\left(\omega \right)$ and $E_{substrate}\left(\omega \right)$ are shown in the inset in Figure 1. Thus, the optical conductivity of ML hBN can be attained by using the Tinkham relation [31]:

(1) $$\frac{E_{sample+substrate}\left(\omega \right)}{E_{sample}\left(\omega \right)}=\frac{1+n}{1+n+Z_{0}\sigma \left(\omega \right)},$$

where *n* is the refractive index of the substrate, $Z_{0}\approx 377$ is the impedance of the free-space, and $\sigma \left(\omega \right)=\sigma_{1}\left(\omega \right)+i\sigma_{2}\left(\omega \right)$ is the complex optical conductivity with ω being the photon frequency. We took n=3.4 for SiO${}_{2}$/Si, 1.96 for quartz, 1.66 for PET, and 3.07 for sapphire to obtain the corresponding optical conductivity. Figure 2 shows the spectra of the real and imaginary parts of σ(ω) for ML hBN on different substrates at different temperatures. As we can see, $\sigma_{1}\left(\omega \right)$ in these samples did not decrease monotonously with increasing frequency, and $\sigma_{2}\left(\omega \right)$ could become negative for hBN on these substrates. Because the THz photon energy (f=ω/2π = 1 THz = 4.13 meV) was much less than the band gap of ML hBN (larger than 6 eV), THz TDS here measured mainly the optical response of heavy holes within the valance band in p-type ML hBN. Hence, we could employ the optical conductivity obtained theoretically for free-electrons to analyze the obtained experimental results. It should be noted that the THz optical conductivity has been widely studied theoretically by using various models such as the series sequence of the free-tunneling carrier transport model [32], the Drude–Lorentz model [33], the Monte Carlo simulation based on the thermal motion of carriers in nanoparticles [34], the Drude–Smith model [34,35], etc. The simplest optical conductivity for free-electrons in an electron gas system is the Drude formula [34]:

(2) $$\sigma \left(\omega \right)=\frac{\sigma_{0}}{1-i\omega \tau }=\frac{\sigma_{0}}{1+{\left(\omega \tau \right)}^{2}}\left(1+i\omega \tau \right),$$

where $\sigma_{0}=e^{2}n_{e}\tau /m^{*}$ is the DC conductivity, $n_{e}$ is the hole density in an ML hBN film, τ is the electronic relaxation time, and $m^{*}$ is the effective hole mass in ML hBN. The conventional Drude formula given by Equation (2) suggests that $\sigma_{1}\left(\omega \right)$ should decrease with increasing ω and $\sigma_{2}\left(\omega \right)$ should always be positive. The features of $\sigma_{1}\left(\omega \right)$ and $\sigma_{2}\left(\omega \right)$ shown in Figure 2 for ML hBN on different substrates cannot be described correctly by the conventional Drude formula given by Equation (2). In this study, we applied the Drude–Smith model [35] for the fitting of the real and imaginary parts of the optical conductivity of ML hBN obtained experimentally, which reads:

(3) $$\sigma \left(\omega \right)=\frac{\sigma_{0}}{1-i\omega \tau }\left[1+\frac{c}{1-i\omega \tau }\right],$$

where the coefficient c=[−1,0] denotes the electronic localization factor induced by a collision between a conducting hole and a scattering center due to the backscattering mechanism with a Poisson distribution [35]. It should be noted that we were able to achieve a good fitting between experimental and theoretical results by taking only the first collision term in the Drude–Smith formula (see Figure 3). Thus, we considered only the first term of the general Drude–Smith formula, namely only one collision event for electronic backscattering was taken into account in Equation (3). Figure 3 shows the experimental and fitted results of the real and imaginary parts of optical conductivity for ML hBN on different substrates at a temperature 280 K. Similar results could be obtained at other measured temperatures. Since the ML hBN used in this study was p-type, we took the effective hole mass of hBN as $0.47m_{0}$ [36,37] in the fitting, where $m_{0}$ is the rest electron mass. Through fitting the experimental results with the Drude–Smith formula, we could obtain the key sample and material parameters such as the hole density $n_{e}$, the electronic relaxation time τ, and the electronic localization factor *c*.

**Figure 4:**
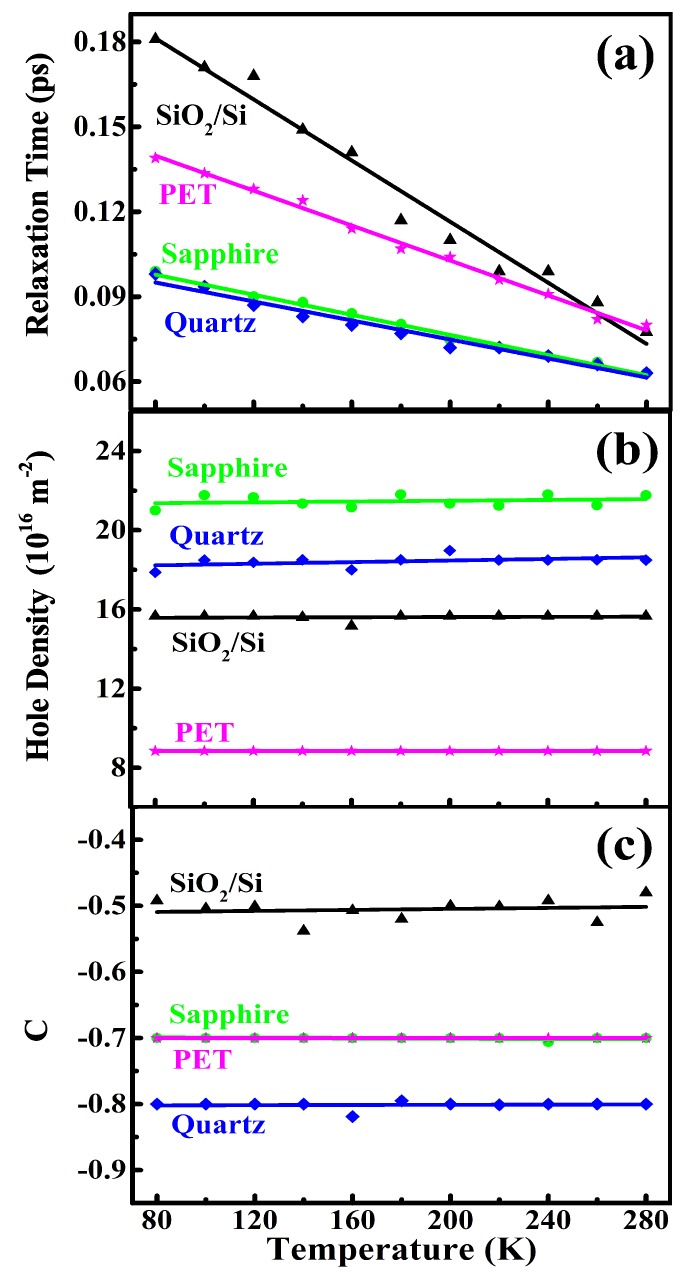
(**a**) Electronic relaxation time, (**b**) hole density, and (**c**) electronic localization factor for ML hBN placed on sapphire, quartz, PET, and SiO${}_{2}$/Si substrates as a function of temperature from 80 K to 280 K.

In the presence of electronic localization or the backscattering effect, the static or DC conductivity could be obtained via $\sigma \left(\omega \to 0\right)\to \sigma_{0}\left(1+c\right)$. Therefore, the effective hole density $n_{e}^{*}$ and the effective electronic relaxation time $\tau^{*}$ could be obtained as $n_{e}^{*}\tau^{*}=n_{e}\tau \left(1+c\right)$. Because c=[0,−1] and τ depends relatively weakly on the backscattering effect [35], the static or effective hole density $n_{e}^{*}$ in ML hBN was less than $n_{e}$. If we assumed $\tau^{*}∼\tau$, we obtained $n_{e}^{*}∼n_{e}\left(1+c\right)$. In Figure 4, we show τ, $n_{e}$, and *c* as a function of temperature for ML hBN on different substrates. From Figure 4, we find that the electronic relaxation time for ML hBN on different substrates decreased with increasing temperature, which is a typical feature of the semiconductor due to carrier-phonon scattering [38]. It is known that the scattering rate for carrier-phonon coupling increases with temperature mainly due to phonon occupation number $N_{0}={\left(1-e^{\hbar \omega_{0}/k_{B}T}\right)}^{-1}$ with $\hbar \omega_{0}$ being the phonon energy. From Figure 4, we note that the hole density $n_{e}$ and the electronic localization factor *c* in ML hBN depended weakly on temperature for four different substrates.

The results shown in Figure 4 indicated that when placing ML hBN on different substrates, different electronic properties could be measured and observed. Similar to a semiconductor based electronic device, the influence of the substrate on the electronic properties of ML hBN was achieved mainly via dielectric ionization, lattice mismatch, proximity-induced interactions, extra scattering centers from the substrate, etc. Below, we discuss these effects in conjunction with the results shown in Figure 4.

(1) Normally, the ML hBN grown by CVD has N-vacancies, which play the role of deep-level impurities [29] and can provide conducting carriers in ML hBN. When low resistance ML hBN prepared in this study was placed on a dielectric substrate, the impurities in the substrate could be ionized, and the holes could be transferred into the hBN layer, because the valance band of ML hBN was much lower than that in a substrate. Thus, the hole density in ML hBN increased in the presence of the dielectric substrate. Similar to a conventional semiconductor on a substrate, the carrier density in ML hBN depends weakly on temperature, as shown in Figure 4b.

(2) The presence of the substrate could provide extra scattering centers, such as impurities [39] and phonons [40], to carriers in ML hBN. When ML hBN was on a substrate, some charges in the substrate could be transferred into ML hBN due to different dielectric constants and electric potentials at the interface. Thus, impurity-like scattering centers could exist in the substrate. For a substrate with a lattice structure, phonon scattering always exists in the substrate, where different substrates have different phonon modes. In Figure 4a, we see that the electronic relaxation time τ differed for ML hBN on different substrates. This was mainly due to the fact that different substrates had different types and strengths of the electronic scattering centers (e.g., impurities and phonons). It is interesting to note from Figure 4a that in the low-temperature regime, τ differed markedly for ML hBN on different substrates. However, at high temperatures, the difference of τ for ML hBN on different substrates became smaller. This implied that at high temperatures, the electronic scattering came mainly from the hBN layer, so that τ depended weakly on the substrate. It should be noted that once τ was obtained, the carrier mobility could be determined by $\mu =e\tau /m^{*}$.

(3) One of the major advantages of the THz TDS measurement was that we could obtain the information of the electronic localization of an electronic system, which could not be directly observed in the electric transport experiment. Because this effect was induced optically via electronic backscattering due to the collisions of electrons distributed randomly with an average time τ [35], the factor *c* in Equation (3) depended sensitively on electronic relaxation time τ or scattering rate 1/τ. Normally, the larger the scattering rate is, the larger the *c*-factor is, where c=[−1,0]. This feature is roughly reflected in Figure 4c.

(4) The lattice mismatch between the substrate and the material could also play an important role in the electronic characteristics of an electronic device. ML hBN is an in-plane hexagonal crystal. SiO${}_{2}$/Si and quartz substrates have tetrahedral and triangular lattice structures, while PET has a polymer structure. Although sapphire and hBN have the same hexagonal structure, sapphire has a lattice constant a=0.476 nm, and ML hBN has a smaller lattice constant a=0.251 nm. Therefore, there exists a lattice mismatch between ML hBN and these substrates. It is known that the lattice mismatch can cause mechanical strain, which can affect the electronic band structure of ML hBN [41,42]. The presence of the lattices mismatch at the interface can also induce the effective electric field perpendicular to the interface, which can enhance the Rashba SOC in the electronic structure of ML hBN [17,19]. Furthermore, due to the presence of lattice mismatch, surface charge carriers can exist at the interface between ML hBN and the substrate. As a result, surface charge density increases as the lattice mismatch between ML hBN and the substrate increases.

(5) SiO${}_{2}$/Si is a commonly and popularly used dielectric substrate material in electronics and optoelectronics. Quartz, sapphire, and PET are often taken as the substrates for optical devices due to the relatively high transmission coefficients especially in the THz regime. From Figure 4, we found that ML hBN on the SiO${}_{2}$/Si substrate had the longest τ and the weakest electronic localization effect along with relatively high hole density, suggesting that SiO${}_{2}$ was a preferable substrate for ML hBN based electronic and optoelectronic devices, compared to other substrates used in this study.

## 4. Conclusions

In this study, we fabricated the low electric resistance ML hBN on different substrates, such as SiO${}_{2}$/Si, quartz, PET, and sapphire. By employing the THz TDS technique, we measured the complex optical conductivity for these samples in the temperature regime from 80 K to 280 K. It was found that the real and imaginary parts of the optical conductivity obtained experimentally for ML hBN on different substrates could fit the Drude–Smith formula well. Thus, we optically determined the key sample and material parameters such as the electronic relaxation time or mobility, carrier density, and electronic localization factor for ML hBN on different substrates. We examined the temperature dependence of these parameters and found that the basic electronic properties of ML hBN depended sensitively on the choice of the substrate. In particular, when ML hBN was placed on these substrates and under THz irradiation, the carrier density could be enhanced considerably, and ML hBN could become conducting. The results obtained from this study suggested that the SiO${}_{2}$/Si substrate was appropriate for ML hBN based electronic and optoelectronic devices, compared to quartz, PET, and sapphire. Furthermore, we demonstrated that THz TDS was a powerful experimental technique in studying and optically characterizing atomically thin electronic systems such as ML hBN.

It is a fact that when applying ML hBN in practical devices for UV, neutron and proton detections and for fabricating the van der Waals heterostructures, it has to be placed on the substrate. The results obtained and discussed in this study could therefore shed some light on the application of ML hBN as advanced electronic and optoelectronic devices. We believe that the results presented in this article could help us to gain an in-depth understanding of the electronic and optoelectronic properties of monolayer hBN.

## Abbreviations

ML	Monolayer
hBN	Hexagonal boron nitride
2D	Two-dimensional
Si	Silicon
SiO${}_{2}$	Silicon dioxide
PET	Polyethylene terephthalate
THz	Terahertz
TDS	Time-domain spectroscopy
UV	Ultraviolet
LEDs	Light-emitting diodes
TMD	Transition metal dichalcogenide
PL	Photoluminescence
SOC	Spin-orbit coupling
CVD	Chemical vapor deposition
PMMA	Poly methyl methacrylate
MOCVD	Metal organic chemical vapor deposition
